# Performance of Sonoelastography for predicting malignancy in soft tissue

**DOI:** 10.1186/s12885-022-10300-4

**Published:** 2022-12-13

**Authors:** Sylvain Bodard, Louis Lassalle, Frédérique Larousserie, Sylvain Guinebert, Thomas Hacquart, Raphael Campagna, David Biau, Noreddine Regnard, Antoine Feydy

**Affiliations:** 1 AP-HP, Hôpital Cochin, Service de Radiologie B, F-75014 Paris, France; 2 AP-HP, Hôpital Necker Enfants Malades, Service d’Imagerie Adulte, F-75015 Paris, France; 3 Université de Paris Cité, F-75006 Paris, France; 4 Sorbonne Université, CNRS, INSERM, Laboratoire d’Imagerie Biomédicale, F-75006 Paris, France; 5 AP-HP, Hôpital Cochin, Service de Pathologie, F-75014 Paris, France; 6 CHU Clermont-Fd, Service de Chirurgie orthopédique et traumatologique, F-63000 Clermont Ferrand, France; 7 AP-HP, Hôpital Cochin, Service d’Orthopédie, F-75014 Paris, France

**Keywords:** Elastography, Soft-tissue masses, Fatty masses, Ultrasonography

## Abstract

**Background:**

Separating benign from malignant soft-tissue masses often requires a biopsy. The objective of this study was to assess whether shear-wave elastography (SWE) helped to separate benign from malignant soft-tissue masses.

**Methods:**

In 2015–2016, we prospectively included patients with soft-tissue masses deemed by our multidisciplinary sarcoma board to require a diagnostic biopsy. All patients underwent ultrasonography (US) followed by SWE to measure elasticity. We compared benign and malignant tumors, overall and after separating tumors with vs. without a fatty component. The biopsy findings, and surgical-specimen histology when available, served as the reference standard.

**Results:**

We included 136 patients, 99 with non-fatty and 37 with fatty soft-tissue masses. Mean elasticity and tumor-to-fat elasticity ratio (T/F) values were significantly lower for the benign than the malignant soft-tissue masses in the overall cohort (30.9 vs. 50.0 kilopascals (kPa), *P* = 0.03; and 2.55 vs. 4.30, *P* = 0.046) and in the non-fatty subgroup (37.8 ± 31.9 vs. 58.9 ± 39.1 kPa, *P* = 0.049 and 2.89 ± 5.25 vs. 5.07 ± 5.41, *P* = 0.046). Data for fatty tumors were non relevant due to lack of conclusive results. By receiver operating characteristics curve analysis, a T/F cutoff of 3.5 had 46% sensitivity and 84% specificity for separating benign and malignant soft-tissue masses.

**Conclusions:**

SWE had good specificity and poor sensitivity for separating benign from malignant soft-tissue masses.

## Key points

- SWE had poor sensitivity for separating benign from malignant soft-tissue masses
- Shear-wave elastography (SWE) results vary with tissue structure
- SWE would not seem capable of decreasing the need for biopsies

## Background

Among soft-tissue masses, over 90% are benign (1, [1], with lipoma being the most common histological type. Soft-tissue sarcomas are uncommon, representing less than 1% of newly diagnosed cancers in the USA [2]. Differentiating benign from malignant soft-tissue masses based on imaging study findings is challenging. In addition, many soft-tissue tumors are classified into intermediate lesions with a potential for local aggressiveness but little propensity for distant dissemination in the 2020 WHO classification. Combining patient characteristics with imaging features can help to place soft-tissue masses on the benign-to-malignant spectrum [3]. Some malignant soft tissue masses are not sarcomas but metastases of carcinoma or of melanoma, or lymphomas [4].

Malignancy should be suspected if the tumor is large, deep, heterogeneous, extensive into neighboring structures, and devoid of clearly benign features (e.g., isolated cyst) [5]. The definitive diagnosis, however, usually requires a biopsy. For instance, lipomas that contain not only fat, but also other tissue components cannot be confidently separated from atypical lipomatous tumors based on the proton density fat fraction computed by magnetic resonance imaging (MRI), fatty acid composition, or texture analysis [6]. To decrease the need for biopsies in patients with soft-tissue masses, new imaging techniques such as functional MRI, ultrasound methods, and artificial-intelligence analysis are being studied. Among them, ultrasound elastography to assess tissue stiffness has shown promise. US elastography is an imaging technique that can show the biomechanical properties of the tissue being examined [7]. There are two US elastography techniques: Strain Elastography (SE) and shear wave elastography (SWE) [7]. In SE elastography user-dependent manual compression is applied to the tissue area under examination while an acoustic impulse is delivered to generate shear waves in SWE which does not require a reference tissue [7]. Thus, SWE allows quantitative measurement [8], is less operator-dependent and is easier to implement [7]. It is currently used as an adjunct to conventional grey-scale ultrasound in routine clinical evaluation [9]. In this technique, the displacement generated by the shear waves that develop perpendicular to the acoustic radiation force pulse applied to the tissue is calculated using a speckle tracking algorithm [7, 10]. It displays the average value of the region of interest (ROI) with a single measurement [9]. Shear waves travel faster through stiffer tissues than through more lax tissues [8]. SWE has been reported to help in characterizing masses in the breast [11], liver [12], prostate [13], and thyroid [14]. Although SWE has also been investigated for evaluating musculoskeletal masses [15], the largest study found no additional benefit of SWE over conventional ultrasonography (US) [16]. In one study, however, the diagnostic accuracy of SWE varied with mass location and patient age [17].

The objective of this single-center prospective observational study in patients with soft-tissue masses deemed to require a biopsy was to determine the diagnostic performance of SWE in differentiating benign from malignant tumors. The hypothesis tested was that malignant lesions are harder than benign ones. The study’s overall goal was to improve patient comfort by reducing unnecessary biopsies and increasing the speed of diagnosis.

## Methods

The study was approved by our institutional review board (IRB: CRM-2112-217). Written informed consent was obtained from each patient before study inclusion.

### Patients

We included consecutive adults with soft-tissue masses deemed by the multidisciplinary sarcoma board at our institution (Cochin University Hospital, Paris, France) composed of radiologists, orthopedic surgeons, oncologists, radiotherapists and anatomopathologists, to require an ultrasound-guided biopsy, between May 2015 and October 2016. Criteria considered eligible for discussion at the sarcoma board are any soft tissue lesions whose benign nature is not obvious or suspicious of malignancy. Patients under the age of 18, and who did not undergo both SWE measurements and a biopsy were not included.

### Ultrasonography (US) and shear-wave elastography (SWE)

US and SWE examinations were performed immediately before the biopsy by one of two musculoskeletal radiologists (with 12 and two years of experience in musculoskeletal radiology, respectively), using a 14-MHz linear transducer and an APLIO 500 (Canon Medical Systems Corporation, Otawara, Tochigi, Japan). The radiologists who performed the SWE, ultrasound, and biopsy had clinical information about the patients, such as pain, and were aware of other imaging findings available in the patients. The patients were in relaxed positions to ensure that no muscle contractions or tissue stretching altered the elastography findings.

US was used to measure tumor size (< 5 cm or > 5 cm) and distance from the skin (in mm) and to assess tumor echotexture (hypoechoic, isoechoic, or hyperechoic) relative to the muscle echogenicity whether the tumor is surrounded by skeletal muscle or subcutaneous fat; the presence of necrosis; and whether the tumor was superficial or deep to the deep fascia. The patient was placed in decubitus position (dorsal, lateral or ventral depending on the location of the tumor), immobile, in apnea if necessary. The ultrasound probe was kept parallel to the skin, under gentle pressure, immobile, under visual control. A round and regular region of interest (ROI) was placed after a 2–3 second delay for signal stabilization and an SWE map was then created. Settings included a minimum depth, a Qbox size adapted to the lesion of at least 10 mm, and a hardness scale of 0 to 40 kilopascals (kPa). The amount of gel placed between the probe and the skin was sufficient to prevent any tissue distortion due to pressure transmitted through the probe. SWE was performed on the most solid and necrosis-free portion of the mass. In case of existing liquefaction or calcification, these areas have been avoided. Shear-wave quality maps were also established and used for the shear-wave velocity (SWV) measurements. The images were stored on the local hard drive of the US machine. We estimated elasticity (in kPa) and the tumor-to-fat elasticity ratio (T/F) between the tumor and the surrounding adipose tissue. We used this ratios to normalize the measurement and try to increase the reproducibility of measurements. We used it even when the tumor was located in the muscle.

The quality of the image was evaluated using at least two of the three following quality controls: the mapping pattern, the shear wave propagation map and the confidence map. About the mapping pattern, the SWE-mapping patterns A and B according to Lino et al. [18] were determined as the acceptable quality, respectively a whole coloring over the tumor/non-tumor lesion (pattern A) and a presence of partial coloring area (larger than half of the target lesion, major color-displayed area in the target lesion ≥50% or more) and small isolated coloring spots (major color-displayed area in the target lesion < 50%) and uncolored areas in the other parts (pattern B). According to propagation quality, “Excellent propagation”, with linear contour lines with equally spaced intervals and “fair propagation”, with some of the lines showing unequally spaced and/or non-linear appearance were determined as the acceptable quality [19]. The confidence map had to be homogeneous.

### Final diagnosis

After local anesthesia, all the tumors were biopsied percutaneously under US guidance by a musculoskeletal radiologist, using a 14-gauge biopsy needle (Achieve, Merit Medical, South Jordan, UT). At least five cores were collected from the areas most likely to be malignant, taking care to avoid cystic components. A senior musculoskeletal pathologist (10 years of experience) routinely examined the biopsy samples, as well as the surgical specimens when available. The search for MDM2 gene amplification was systematically performed. These histological results served as the reference standard against which the diagnostic performance of SWE was evaluated.

### Statistical analysis

The statistical analysis was conducted using SPSS software version 20 (SPSS, Chicago, IL). Quantitative study variables were described as mean ± SD or median and interquartile range (IQR) for non normally distributed variables and qualitative binary variables as n (%). For comparisons of the groups with a final diagnosis of benign and malignant masses, we chose the student t test for quantitative variables and the chi-square test for binary variables.

To assess the diagnostic performance of SWE, we plotted receiver operating characteristics (ROC) curves to determine the tumor elasticity and T/F cutoffs that maximized Youden’s index. We computed the sensitivity and specificity of elasticity and T/F using the optimal cutoffs, with their 95% confidence intervals (95%CIs).

We then separated the tumors in the benign and malignant groups into subgroups depending on whether fatty tissue was present within the mass. Fat-containing tumors have lower elasticity than other tissue lesions and there are benign (lipomas) and malignant (liposarcomas) fatty tumors just as there are benign and malignant non-fatty tumors. We repeated the analyses for these subgroups.

## Results

### Study population and tumors

**Fig. 1:**
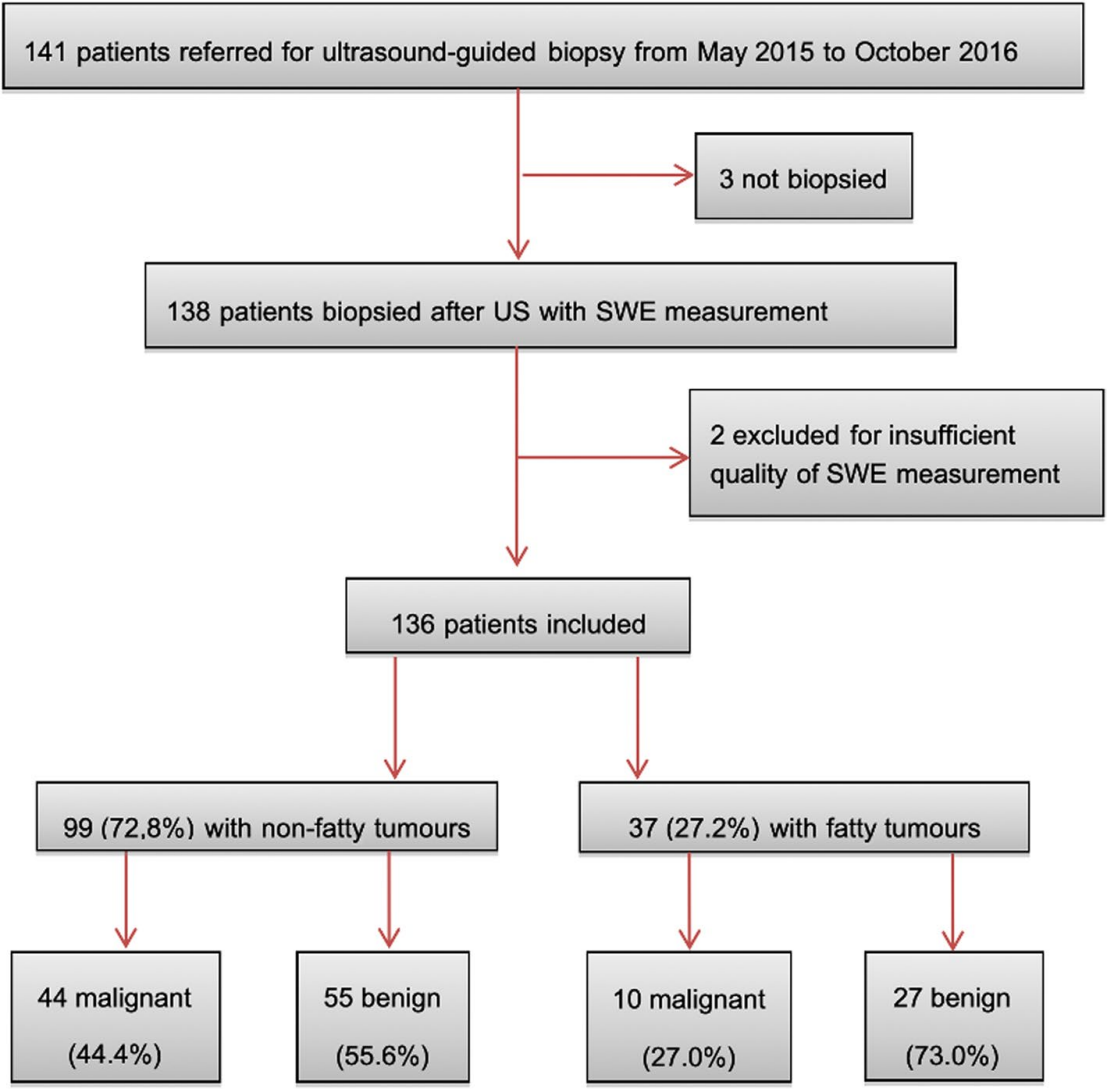
Flowchart of study participants

Figure 1 is the study flow chart. Of the 136 included patients, 99 had non-fatty tumors (44 malignant and 55 benign) and 37 fatty tumors (10 malignant and 27 benign). There were 69 (50.7%) males for 67 (49.3%) females. There was no significant gender difference between benign lesions (38 males (27.9%) for 44 females (32.4%)) and malignant lesions (31 males (22.8%) for 23 females (16.9%)) (*p* = 0.207). The mean age of the patients was 49.3 years (range, 18–86). There was no significant difference. 104 (76.5%) tumors were deep and 32 (23.5%) superficial. Among the benign lesions, 59 (71.9%) were deep, and 23 (27.9%) were superficial. Among the malignant lesions, 45 (83.3%) were deep and 9 (16.7%) superficial. There was no significant difference in topography between benign and malignant lesions (*p* = 0.13). The median maximum diameter of the lesions was 64 mm (IQR: 37–89 mm). Malignant lesions had a significantly larger diameter than benign lesions (median: 76 mm, IQR: 41–99 mm) versus (median: 59 mm, IQR: 36–83 mm) (*p* = 0.046). The median depth of the lesions was 8 mm (IQR: 4–11 mm). There was no significant difference in depth between benign (median: 8 mm, IQR: 4-12 mm) and malignant (median: 10, IQR: 5–13) lesions (*p* = 0.23).

**Fig. 2:**
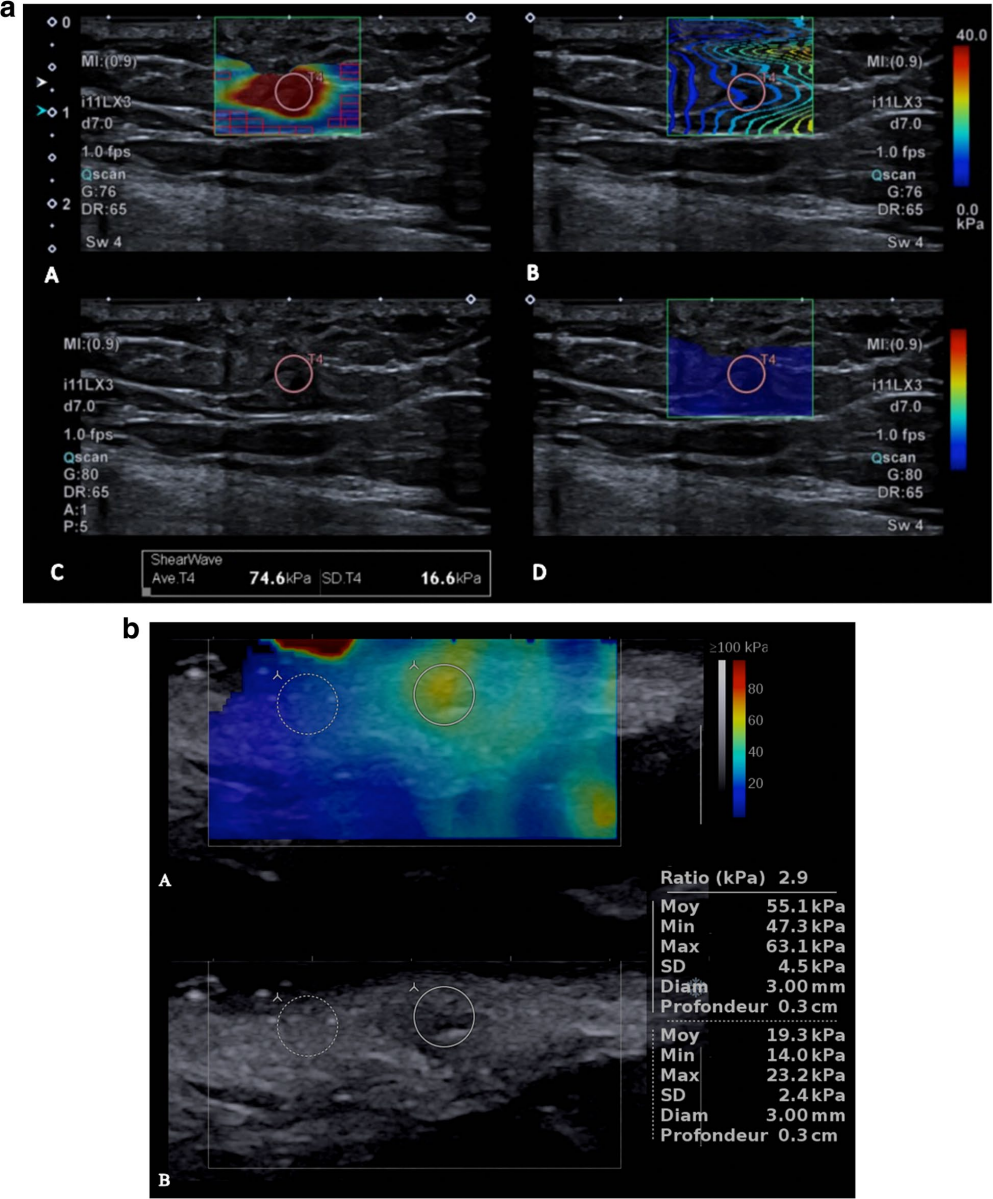
a. 64-year-old patient with a benign subcutaneous mass (lipoma) of intermediate hardness. Elasticity map (A), shear wave propagation map (B), B-mode map (C), confidence map (D). b. 65-year-old patient with a pulmonary adecarcinoma metastasis in subcutaneous fat exhibiting a hight level of hardness Elasticity map (A), B-mode map (B)

Table 1 reports the main features of the patients and masses and Table 2 the histological diagnoses. The histological diagnosis was not determined for one lesion, which was certainly benign (either spindle-cell lipoma or elastofibroma). Figure 2a and b show representative examples of a benign tumor (lipoma) and malignant tumor (pulmonary adecarcinoma metastasis). Note the final diagnoses for the tumors included in this study are up to date with the most recent WHO classification.

**Table 1:** Features of the 136 patients and tumors

	Overall cohort	Benign	Malignant	*P* value
Number of patients, n (%)	136	82 (60.3)	54 (39.7)	–
Males/Females, n (%)	69/67 (50.7/49.3)	38/44 (27.9/32.4)	31/23 (22.8/16.9)	0.207
Age, y, mean ± SD	49.3 (range16–86)	47.5 ± 17.3	51.9 ± 17.5	0.15
Location: deep/superficial, n (%)	104/32 (76.5/23.5)	59/23 (71.9/28)	45/9 (83.3/16.7)	0.13
Maximum diameter, mm, median (IQR)	64 (37–89)	59 (36–83)	76 (41–99)	**0.046**
Distance from the skin, mm, median (IQR)	8 (4–11)	8 (4–12)	10 (5–13)	0.23
Well-defined margins, yes/no, n (%)	125/11 (91.9/8.1)	75/7 (91.5/8.5)	50/4 (92.6/7.4)	0.81
Multilobular, yes/no, n (%)	37/99 (27.2/72.8)	21/61 (25.6/74.4)	16/38 (29.6/70.4)	0.61
Necrosis,^b^ yes/no, n (%)	20/115 (14,7/84,5)	9/72 (10,9/87,8)	11/43 (20,3/79,6)	0.109
Echotexture hypoechoic, n (%)	65/71 (47.8/52.2)	36/46 (43.9/56.1)	29/25 (53.7/46.3)	0.173
Elastography, kPa, median (IQR)	38.1 (22,4–54.6)	32,1(18.2–51,1)	51.2 (31.3–71.0)	**0.004**
T/F^*^, median (IQR)	3.4 (1.2–4.9)	2.34 (1.4–4.7)	4.1 (2.1–6.1)	**0.047**

^*^T/F ratio of elasticity of the tumor over the elasticity of the surrounding fat tissue

^a^available for 134 patients

^b^available for 135 patients

**Table 2:** Histological diagnoses for the 136 soft-tissue masses

	Fatty tumors	Non-fatty tumors
**Benign lesions**	24 lipomas^a^ 1 elastofibroma 1 hibernoma 1 spindle-cell lipoma	5 desmoid tumors 5 hemangiomas 5 schwannomas 6 old hematomas 4 nodular fasciitis 4 solitary fibrous tumors 4 villonodular synovitis^b^ 4 benign nerve sheath tumors 3 non-diagnostic benign-appearing fibrous tissue 3 myxomas 2 chronic abscesses 2 circumscribed ossifying myositis 2 neurofibromas 1 benign myxoid tumor 1 Dupuytren’s disease 1 Ledderhose disease 1 tenosynovitis 1 ulcerated synovitis
**Malignant lesions**	8 atypical lipomatous tumors 1 dedifferentiated liposarcoma 1 myxoid liposarcoma	5 synovial sarcomas^b^ 2 leiomyosarcomas (grade 2 and 3) 3 dedifferentiated pleomorphic sarcomas (2 grade 3, 1 grade 2) 3 epithelioid sarcomas 3 lymphomas (B and Hodgkin) 3 myxofibrosarcomas (2 grade 1 and 1 grade 3) 2 chondrosarcomas (recurrences) 2 extraskeletal myxoid chondrosarcomas 2 Ewing sarcomas (extraskeletal sites) 2 kaposi sarcomas 1 alveolar soft-tissue sarcoma 1 extraosseous ewing sarcomas 1 malignant solitary fibrous tumor 1 myxofibrosarcoma (grade 2) 1 myxoinflammatory fibroblastic sarcoma 1 osteosarcoma (recurrence) 1 pleomorphic hyalinizing angiectatic tumor 1 superficial CD34-positive fibroblastic tumor 8 metastasis (leiomysarcomas, Ewing sarcoma, chondrosarcomas, 2 melanoma, sarcoma, papillary thyroid carcinoma, pulmonary adecarcinoma)

^a^including 5 altered and 1 with inflammatory changes

^b^is not part of the soft-tissue tumors but addressed for suspicion of soft-tissue tumors

### Comparison of benign and malignant tumors

Table 1 compares the main tumor features. Malignant lesions were larger and stiffer and the T/F were significantly higher in the malignant group. None of the other features differed significantly between the groups with benign and malignant lesions.

**Fig. 3:**
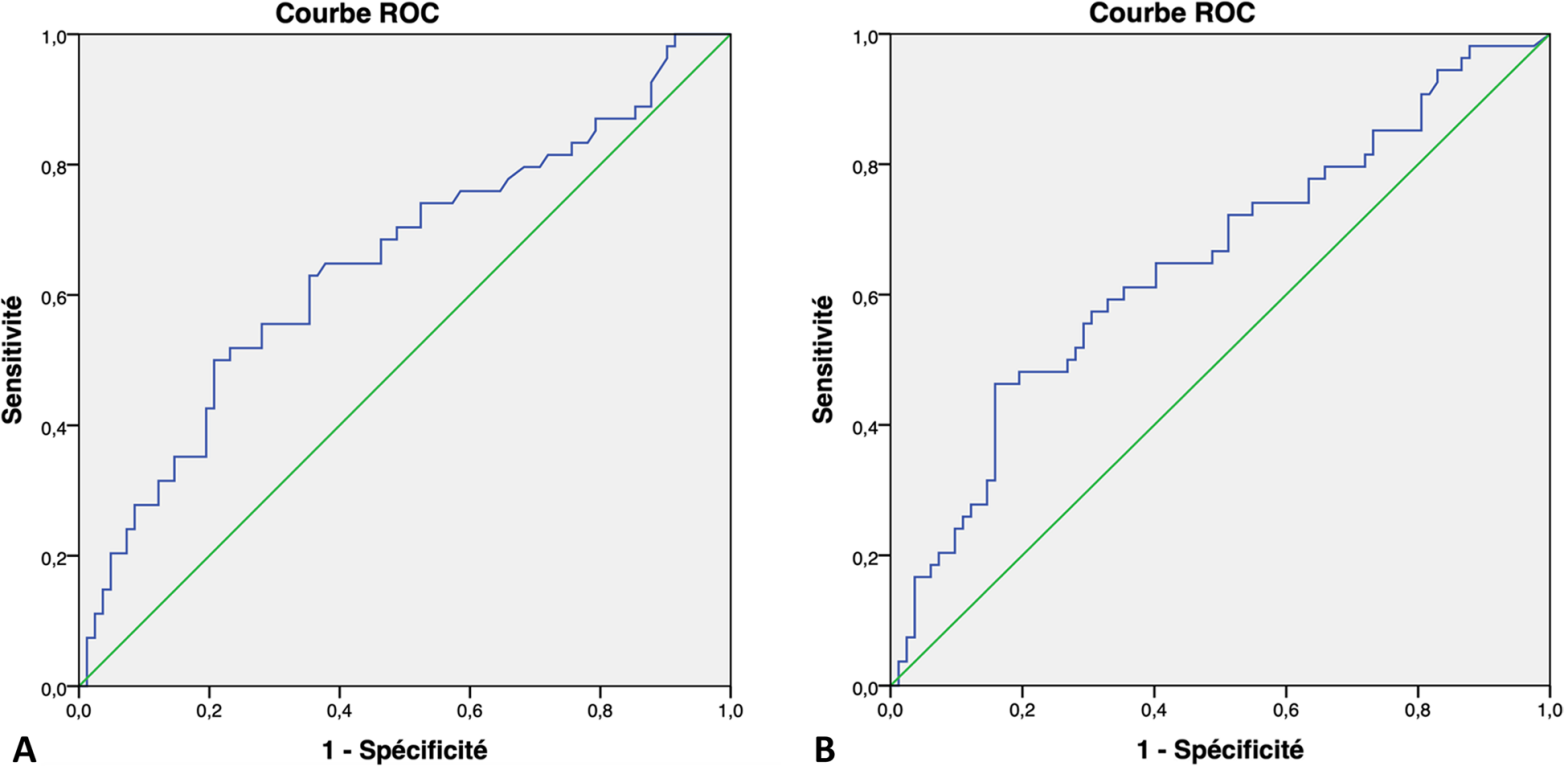
Receiver operating characteristic plots of elasticity (**A**) and elasticity ratio in the tumor over the surrounding fat (T/F) (**B**)

Figure 3 shows the ROC curve analysis. For elasticity, the optimal cutoff was 40.8 kPa, with 50% sensitivity and 79% specificity. The optimal cut-off for T/F was 3.5, with 46% sensitivity and 84% specificity. The areas under the ROC curve were 0.65 (95% confidence interval, 0.55–0.75) for elastography and 0.65 (0.55–0.74) for T/F.

### Study of tumors according to fat content

#### Non-fatty tumors

**Fig. 4:**
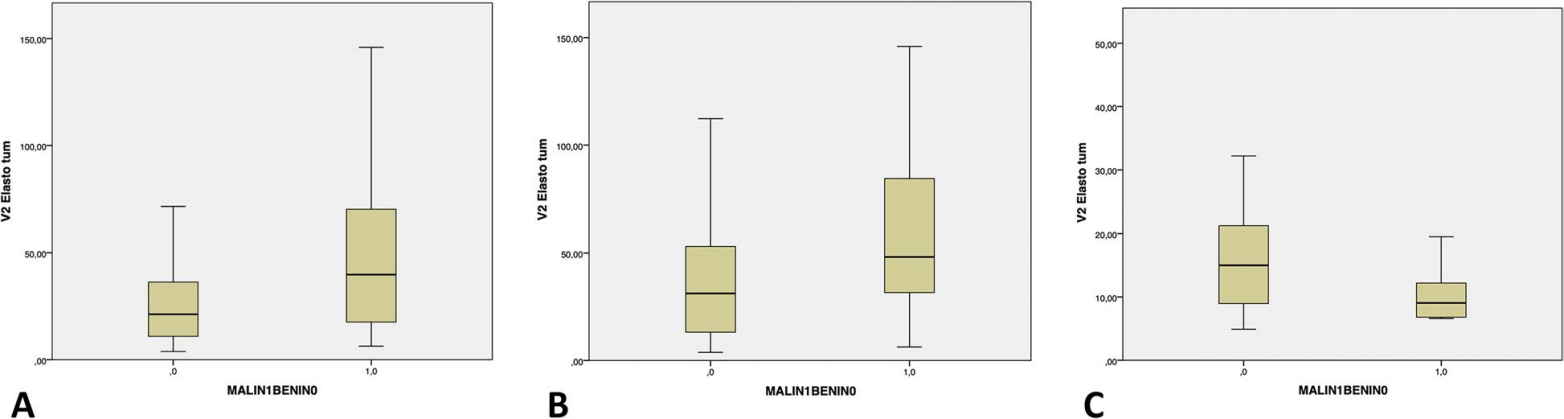
Box plot of the statistical difference in elasticity in the overall cohort (**A**) and in the subgroups with fatty (**B**) and non-fatty tumors (**C**) The boxes indicate interquartile ranges; horizontal lines, the median; and whiskers, extreme values

**Fig. 5:**
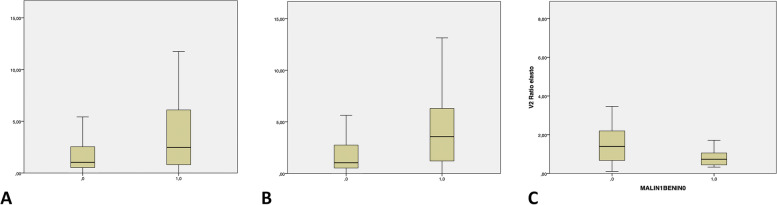
Box plot of the statistical difference in elastography ratios in the overall cohort (**A**) and in the subgroups with fatty (**B**) and non-fatty tumors (**C**). The boxes indicate interquartile ranges; horizontal lines, the median; and whiskers, extreme values

Of the 99 tumors containing no fat, 55 (55.6%) were benign and 44 (44.4%) malignant. Table 3 reports the main features of these tumors. Mean elasticity and mean T/F were significantly higher in the malignant group. Figures 4 and 5 are the box plots of these two parameters.

**Table 3:** Findings for the 99 non-fatty tumors

	Overall cohort	Benign	Malignant	*P* value
Number of patients, n (%)	99	55 (55.6)	44 (44.4)	–
Males/Females, n (%)	55/44 (52.5/47.5)	26/29 (47.3/52.7)	26/18 (59.1/40.9)	0.24
Age, y, mean ± SD	46.6 (range 16–86)	44.9 ± 17.7	49.6 ± 17.4	0.14
Location: deep/superficial, n (%)	72/27 (72.7/27.3)	36/19 (65.4/34.5)	36/8 (81.8/18.2)	0.07
Maximum diameter, mm, median (IQR)	50 (31–68)	44 (27–60)	58 (37–74)	0.08
Distance from the skin, mm, median (IQR)	8 (5–11)	7 (4–10)	9 (4–11)	0.13
Well-defined margins, yes/no, n (%)	91/8 (91.9/8.1)	51/4 (92.7/7.3)	40/4 (90.9/9.1)	0.74
Multilobular, yes/no, n (%)	30/69 (30.3/69.7)	15/40 (27.3/72.7)	15/29 (34.1/65.9)	0.46
Necrosis^b^, yes/no, n (%)	15/83 (15.3/84.7)	5/49 (9.2/90.7)	10/34 (22.7/77.3)	0.06
Echotexture hypoechoic n (%)	60/9 (60.6/39.4)	33/32 (50.8/49.2)	27/17 (61.4/38.6)	0.53
Elastography, kPa, median (IQR)	46.8 (26.8–60.3)	36.7 (21.9–52.1)	59.1 (38.2–78.2)	**0.048**
T/F^*^, median (IQR)	3.6 (1.4–6.1)	2.7 (0.3–5.1)	5.3 (2.7–7.8)	**0.045**

*T/F* ratio of elasticity of the tumor over the elasticity of the surrounding fat tissue

^a^available for 97 patients

^b^available for 98 patients

#### Fatty tumors

Of the 37 fatty tumors, 27 (73%) were benign and 10 (27%) malignant. Table 4 shows their main characteristics. The median maximum diameter was significantly larger in the malignant group. Neither the elasticity nor T/F differed significantly between the benign and the malignant tumors. Figures 4 and 5 are the box plots of these two parameters.

**Table 4:** Features of the 37 fatty tumors

	Overall cohort	Benign	Malignant	*P* value
Number of patients, n (%)	37	27 (73)	10 (27)	–
Males/Females, n (%)	17/20 (45.9/54.1)	12/15 (44.4/55.5)	5/5 (50/50)	0.76
Age, y, mean ± SD	56.2 (range 27–86)	53.9 ± 14.9	62.2 ± 14.5	0.14
Location: deep/superficial, n (%)	32/5 (86.5/13.5)	23/4 (85.2/14.8)	9/1 (90/10)	0.70
Maximum diameter, mm, median (IQR)	105 (71–168)	82 (54–104)	165 (125–198)	**0.017**
Distance from the skin, mm, median (IQR)	11 (7–13)	11 (6–13)	14 (10–14)	0.29
Well-defined margins, yes/no, n (%)	34/3 (91.9/8.1)	24/3 (88.9/11.1)	10/0 (100/0)	0.27
Multilobular, yes/no, n (%)	7/30 (18.9/81.1)	6/21 (22.2/77.8)	1/9 (10/90)	0.40
Necrosis, yes/no, n (%)	5/32 (13,5/86,5)	4/23 (14.8/85.2)	1/9 (10/90)	0.59
Echotexture hypoechoic, n (%)	5/32 (13,5/86,5)	3/24 (11.1/88.9)	2/8 (20/80)	0.41
Elastography, kPa, median (IQR)	14.9 (9.6–17.2)	16.9 (11.5–19.2)	11.3 (5.9–16.0)	0.09
T/F^*^, median (IQR)	1.4 (0.4–2.1)	1.6 (0.9–2.5)	0.9 (0.3–1.7)	0.09

*T/F* ratio of elasticity of the tumor over the elasticity of the surrounding fat tissue

## Discussion

In patients with soft-tissue masses and a definitive histological diagnosis, malignant tumors had significantly higher elasticity and T/F values than did benign tumors. However, sensitivity was limited for both elasticity (50% with a cutoff of 40.8 kPa) and T/F (46% with a cutoff of 3.5). Specificity was higher (79 and 84%, respectively). When we performed a separate analysis of tumors with and without fat components, we found that the differences in elasticity and T/F between malignant and benign tumors were significant only for the tumors devoid of fat.

US and MRI often fail to confidently separate benign from malignant in soft-tissue masses. Malignant tumors have been reported to be harder in consistency than benign tumors, notably breast and thyroid carcinomas [20]. The abundance of stromal fibrous reaction in some carcinomas, for example in breast carcinoma, papillary thyroid carcinoma or pancreatic adenocarcinoma, or the high cell density in some malignant tumors such as prostate and lung carcinomas, combined with the strong attachment of malignant cells to surrounding tumors, may explain this finding [21]. However, in a prospective study of 50 STTs done at a sarcoma center, longitudinal SWV was 30% slower in malignant than in benign tumors, indicating greater softness of the malignancies, and did not vary significantly with age, sex, or tumor size [22]. In soft tissue tumors, cell density is not always proportional to malignancy, unlike in epithelial tumors. The considerable variability in tissue structure of STTs and heterogeneity of mesenchymal tumors in cell density and stroma (absent, fibrous, myxoid, hemorrhagic, highly or poorly vascularized) create diagnostic challenges compared to organs where tumor structure is more uniform, such as papillary thyroid carcinoma. The absence of significant differences in elasticity and T/F in our study may be ascribable to the considerable variability in tissue structure of soft-tissue masses. Thus, an evaluation of 48 superficial STTs showed that epidermoid cysts were stiffer than were ganglion cysts and lipomas, in keeping with their greater hardness to palpation [23]. Moreover, selection bias occurred in our study, as we included only patients deemed eligible for a biopsy. Thus, most patients with benign lipoma-type lesions that did not require further investigation were not studied. The lipomas in our cohort had atypical presentation due to tissular alterations, resulting in diagnostic uncertainty. An earlier study in a smaller sample (*n* = 32) included a high proportion of lipomas and had no hard benign masses such as myositis ossificans [24]. In a feasibility study of 32 superficial STTs, of which 12 were malignant, greater hardness correlated with a greater risk of malignancy [24]. When used with US, SWE improved the discrimination between benign and malignant masses in soft-tissue in a study of 206 tumors, including 79 malignancies, diagnosed by biopsy [17]. The diagnostic contribution of SWE varied according to patient age and to whether the tumor was superficial or deep to the superficial fascia. No elasticity or T/F cutoffs were determined. Ultrasound measurements of mean and maximum SWV for 43 benign and 37 malignant STTs found no significant differences in either parameter [21]. SWV was not associated with the malignant or benign nature of STTs in studies of 105 and 151 tumors [16]. These differences across studies may be due to differences in the structure of the STTs, with variability regarding fibrotic changes, bleeding, necrosis, myxoid changes, calcification, and cystic degeneration [25].

One of the original features of our study is the measurement of the elasticity ratio of the tumor and the surrounding fatty tissue (T/F). However, this parameter did not add meaningfully to the diagnostic performance of SWE. In addition, the border between the mass and the surrounding unaffected tissue can be difficult to assess for infiltrating tumors. Strain elastography has been reported to improve the diagnosis of soft-tissue masses, with a significantly lower strain ratio for benign tumors, although strain histograms and visual scoring showed no significant differences [26].

Maximum median diameter was greater in the malignancies in the overall cohort and the subgroup of fatty tumors. None of the other US features differed significantly between the benign and malignant soft-tissue masses. A study in which experienced musculoskeletal radiologists evaluated 823 STTs showed 81% diagnostic accuracy of US, with 93.3 and 97.9% sensitivity and specificity of US for diagnosing malignancies, respectively [27]. Diagnostic performance increased with radiologist experience. In keeping with our findings, published data indicate that factors associated with malignancy include older age, deeper location, larger size, less sharply defined margins, hypoechoic appearance, and hypervascularity [16, 17, 22, 25, 28]. However, age and lesion depth, well-established criteria for malignancy in soft tissue sarcomas, were not significant discriminators of malignant versus benign lesions in the present study. We believe that this is due to a lack of power.

Our study has several limitations. We used only ultrasonography and SWE: we did not assess the potential diagnostic improvements that might be obtained by adding MRI. Neither the size of the ROI nor its location within heterogeneous masses was standardized. All biopsies were done under US guidance, as opposed to computed tomography guidance, but this method constitutes standard practice. Indeed, CT is not an ideal technique for soft tissue lesions especially if they are superficial and small.

Another limitation concerns the technical and physical properties of the technique itself, especially when evaluating tumors of different depths (distance from the probe) and tumors surrounded by different tissues, such as subcutaneous fat versus muscle, which influence shear wave propagation differently. Moreover, some large tumors might protrude to the surface, which can significantly influence SWE examination. However, in our series, there was no such tumor. Furthermore, the larger tumor might increase the internal heterogeneity echo. When the tumors were heterogeneous, we averaged the hardness.

Finally, we studied only two soft-tissue masses subgroups, with and without fat components, although soft-tissue masses are highly heterogeneous in histological nature and structure. It might therefore be of interest to evaluate the diagnostic usefulness of SWE in subgroups determined by standard US.

## Data Availability

All data are stored on a computer at the Cochin Hospital (AP-HP) and are available. The datasets generated and/or analysed during the current study are not publicly but are available from the corresponding author on reasonable request.

## Abbreviations

SWE: Shear-wave elastography
US: Ultrasonography
MRI: Magnetic resonance imaging
ROI: Region of interest
ROC: Receiver operating characteristics curves
T/F: Tumor over the surrounding fat
